# Qiangjing tablets repair of blood-testis barrier dysfunction in rats via regulating oxidative stress and p38 MAPK pathway

**DOI:** 10.1186/s12906-022-03615-z

**Published:** 2022-05-14

**Authors:** Junjun Li, Yaodong You, Peihai Zhang, Xiaopeng Huang, Liang Dong, Fang Yang, Xujun Yu, Degui Chang

**Affiliations:** 1 TCM Regulating Metabolic Diseases Key Laboratory of Sichuan Province, Hospital of Chengdu University of Traditional Chinese Medicine, Chengdu, 610072 The People’s Republic of China; 2 The Reproductive & Women-Children Hospital, Chengdu University of Traditional Chinese Medicine, Chengdu, 610041 The People’s Republic of China; 3 School of Medical and Life Sciences, Chengdu University of Traditional Chinese Medicine, Chengdu, 611137 The People’s Republic of China

**Keywords:** The blood-testis barrier, Oxidative stress, p38 MAPK pathway, Traditional Chinese Medicine, Qiangjing tablets

## Abstract

**Background:**

The blood-testis barrier (BTB) is a physical barrier of the testis to prevent various exogenous substrates from entering apical compartments and provides immune privilege for spermatogenesis, which is essential for normal spermatogenic function of testis. It has been shown that oxidative stress can damage BTB by activating the p38 MAPK pathway. In Traditional Chinese Medicine, Qiangjing tablets (QJT) improve spermatogenesis and increase pregnancy rates. Previous studies have confirmed that QJT can improve sperm quality and have obvious antioxidant effects. In this study, we explore whether QJT contributes to recovery from BTB dysfunction in rats.

**Methods:**

BTB dysfunction was induced in rats by 1% Cyclophosphamide (CP). The CP-induced rats in the treatment group were given a dose of QJT (0.45 g/kg·d) by gavage. Testis tissues were collected for histopathological and biochemical analysis, and the testis weight was estimated. Levels of BTB-related proteins and antioxidant enzyme were analyzed in the testis tissues.

**Results:**

QJT resolved the pathological injury of rats testis induced by CP. Furthermore, MDA levels were significantly reduced, and the levels of SOD markedly increased in the testicular tissue after QJT treatment. In addition, QJT down-regulated the expression of p38 protein in rat testis and up-regulated the expressions of key proteins ZO-1, occludin and F-actin in BTB.

**Conclusion:**

These results demonstrate that QJT exerts protective effects on CP-induced rats with BTB dysfunction, likely by regulating the oxidative stress-mediated p38 MAPK pathway.

**Supplementary Information:**

The online version contains supplementary material available at 10.1186/s12906-022-03615-z.

## Introduction

Infertility is a social and medical issue of global concern. It is estimated that infertility affects 8–12% of couples globally, with a male factor being a primary or contributing cause in approximately 50% of couples [1]. The decline of sperm quality is an important reason for male infertility. In nearly half of infertility cases, the male partner has suboptimal sperm parameters [2]. There are many factors leading to abnormal sperm quality, among which the damage of blood testicular barrier (BTB) is one of the important factors. The relationship between BTB disfunction and impediment of spermatogenesis was widely confirmed. Damage of the BTB will lead to oligospermia, asthenospermia, teratospermia, sperm DNA damage, and even anti-sperm antibodies resulting in male infertility [3]. The main structure of BTB includes the tight junctions (TJ), which are constituted by cell-adhesion complexes and seal tissues, contributing in cell polarity and signaling. The proteins such as zonula occludens-1 (ZO-1) and Occluding, etc. distributed in TJ play a crucial role in keeping the normal function of BTB [4]. It has been confirmed that oxidative stress is closely related to BTB damage [5] and its induction of BTB injury mediated through a series of signal transduction pathways in the cell. P38 mitogen-activated protein kinase (p38MAPK), a kinase belonging to the MAPK superfamily, which is implicated in oxidative stress response [6]. In the testis, the activated p38 MAPK cascade was implicated in disruption of the tight junction barrier and loss-of-junction proteins [7, 8], resulting in the decline of endocytosis and reproductive function of spermatogenic cells [9] Thus, the activation of the p38 MAPK pathway is one of the mechanisms of oxidative stress-induced BTB injury.

CP is an alkylating agent with oral activity, which is widely used as antitumor and immunosuppressant. Testicular toxicity is one of the main side effects of CP, and oxidative stress damage is one of the main causes of testicular spermatogenesis disorders caused by CP [10]. Moreover, the loss of BTB barrier function induced by CP associates with an increase in p38 phosphorylation [11]. Many studies have shown that substances with antioxidant effects, such as melatonin [12], ostrea rivularis polysaccharide (ORP) [13], boron [14], can inhibit the oxidative damage of testis caused by CP and protect reproductive function. However, there are few studies on the repair of BTB by protective agents through p38 MAPK pathway. Moreover, there is no relevant research on the treatment of CP induced BTB injury by traditional Chinese medicine through p38 MAPK pathway.

Qiangjing tablets (QJT), which are composed of a variety of traditional Chinese medicine, has the functions of improving spermatogenesis and increasing pregnancy rates. It has been used in China to treat male infertility for more than 20 years. Our previous studies have shown that CP may lead to infertility via regulating the Toll-like signaling pathway, decreasing sperm concentration, viability and inducing the damage of testicular spermatogenesis [15]. QJT modified the reproductive function of male rats through reducing the level of Toll-like receptor 4 (TLR4), Myeloid differentiation factor 88 (MyD88) and increasing TNF receptor associated factor 6 (TRAF6) on Toll like signaling pathway in testicular tissue [15]. Meanwhile, QJT may enhance semen quality in azoospermic rats through mediating MAPK signaling pathway against oxidative stress [16]Also, QJT can improve sperm quality by down-regulating Fas/FasL signaling pathways, reducing the apoptosis rate of spermatogenic cells and increasing the expression of Occludion, which is a key protein in the BTB [17, 18].

In summary, the above studies have shown that QJT is a potentially effective drug for male infertility. However, up to now, the mechanisms of QTJ in improving sperm quality are not completely clear. Thus, in this study, we investigate the effect of QJT in mitigating the damage of the BTB in testicular tissue using the CP-induced BTB injury rats model.

## Materials and methods

### Drugs and reagents

The specific formula of QJT is as follows: Ginseng Radix Et Rhizoma (Araliaceae; the root of Panax ginseng C. A. Meyer), Angelica Sinensis Radix (Umbelliferae; the dried root of Angelica sinensis (Oliv) Diels.), Rehmanniae Radix Praeparata (Scrophulariaceae; root tuber from Rehmannia glutinosa Libosch), Corni Fructus (Cornaceae; the pulp of the ripe fruit of Cornus oj-jZcinalis Sieb. etZucc.), Lycii Fructus (Solanaceae; the dried mature fruit of Lycium barbarum L.), Schisandrae Chinensis Fructus (Magnoliaceae; the dried ripe fruit of Schisandra chinensis (Turcz.) Baill.), Cuscutae Semen (Convolvulaceae; the dry mature seed of Cuscuta chinensis Lam), Plantaginis Semen (Plantaginaceae; the dried mature seeds of Plantago asiatica L. or Plantagodepressa Willd.), Epimedii Folium (Berberidaceae; the dried aerial parts of Epimedium Brevicornu Max-Im.), Common Curculigo orchioides (Amaryllidaceae; the rhizome of Curculigo orchioides Gaertn.), Herba Leonuri (Lamiaceae; the overground part of Leonurus japonicus Sweet). The above traditional Chinese medicines were purchased from Sichuan Traditional Chinese Medicine Group Co., Ltd (Chengdu, China). The use of all the above traditional Chinese medicines complies with the drug administration law of the people's Republic of China and the law of the people's Republic of China on traditional Chinese medicine. After appropriate grinding, they were extracted by reflux with 10 times and 8 times water respectively, 1 h each time, filtered, combined with the two kinds of filtrates, concentrated to 1 g/ ml under reduced pressure. Then methanol was added according to the volume ratio of 2:1 and left overnight. Take 100 ml supernatant, add methanol at 1:1, centrifugate at 3,000 r/ min for 3 min, and collect the supernatant. Take 1–5 ml supernatant and filtrate with 0.45 μm microporous membrane.

Reference solution: ferulic acid, Hyperoside, geniposide, loganin, verbascoside, morroniside, ginsenoside Re, Ginsenoside Rg1, schisandrin A, curculioside, stachydrine and motherwort alkaloid, and 0.1 mg of each Chinese medicine. Put them in a 10 ml volumetric flask, add methanol, and then use ultrasonic dissolution. Constant volume and obtain the mixed reference solution.

In order to provide a reliable method for the quality control of QJT, fingerprints of QJT and reference solution were compared in our previous study. At the same chromatographic conditions, there were 12 common peaks in fingerprints of both QJT and reference solution, which showed that the type or content of QJT appeared to no change greatly before and after preparation [19].

Cyclophosphamide for Injection (Lot.No.16091325) was obtained from Jiangsu Shengdi Pharmaceutical Co., Ltd.(Jiangsu, China). 0.9% sodium chloride (Lot.No.D150426021) was ordered from Shandong Kelun Pharmaceutical Trading Co., Ltd. (Shandong, China). pHam’s F-10 culture medium (Lot.No.0901A20) was procured from Zhejiang Lianshuo Biological Technology Co. Ltd. (Zhejiang, China). Normal goat serum (Lot.No.PH0424) was procured from Fuzhou Phygene Biological Technology Co. Ltd. (Fuzhou, China). MDA (Lot.No.ml077384) and SOD (Lot.No.ml059387) kits were obtained from Shanghai Jianglai Biotechnology Co., Ltd.(Shanghai, China). Goat anti-mouse IgG H&L (AlexaFluor4®88) (Lot.No.ab150113), Goat anti-rabbit IgG H&L (HRP) (Lot.No.ab205718), Prism Ultra Protein Ladder (Lot.No.ab116028), DAB kit (Lot.No.ab64238), Fluoroshield sealant (Lot.No.ab104139), Anti-P38 (Lot.No.ab170099), Anti-F-actin (Lot.No.ab205), Anti-Occludin (Lot.No.ab216327), and Anti-ZO1 (Lot.No.ab221547) were procured from Abcam Plc. (Cambridge, UK). Clarity Western ECL Substrate (Lot.No.1705060) and Immun-Blot PVDF Membrane (Lot.No.1620177) were ordered from Bio-Rad Laboratories (Shanghai) Co., Ltd. (Shanghai, China).

### Experimental animals and medicinal intervention

Male Sprague Dawley (SD) rats (180–220 g, 4–7 weeks old) were purchased from Chengdu Dossy Experimental Animal Co., Ltd. (Chengdu, China). The rats were reared in environmentally controlled conditions with temperature of 22 ± 2 °C, relative humidity of 40%-70%, light/dark cycle for 12 h, and had free access to water and food. All experiments were administrated and approved by the Ethics Committee of Chengdu University of Traditional Chinese Medicine (Ethics committee approval document number: 2021DL-014).

**Fig. 1:**
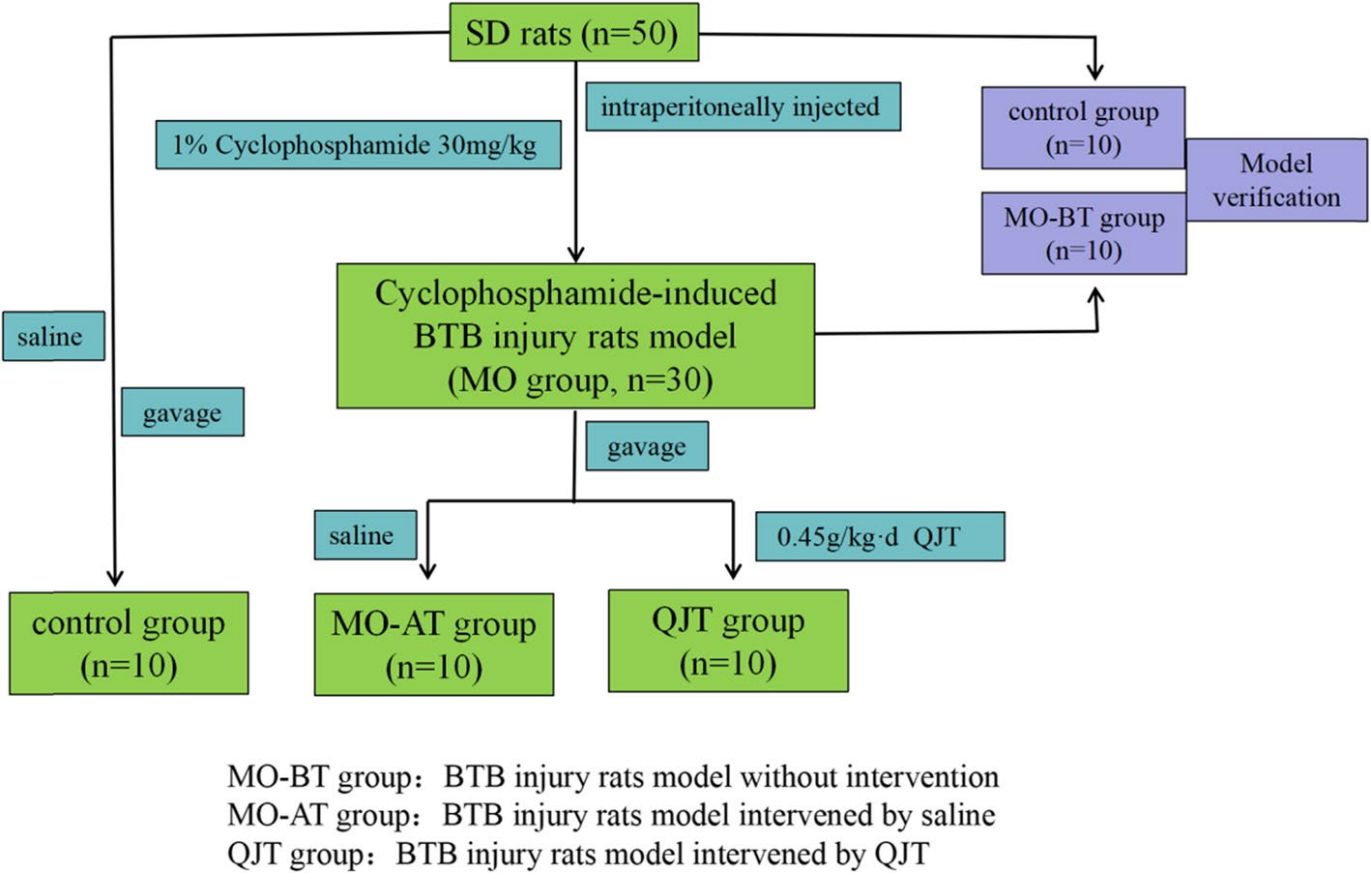
Experimental process.Healthy rats were injected with CP daily for 5 days. Eight days after the first CP dose, CP-induced rats received QJT or the saline via intragastric administration. After the QJT treatment for 4 weeks, testicular tissues and function were evaluated

A total of Fifty SD rats were included in this study. After 3 days of adaptive feeding, 30 rats were randomly selected and injected intraperitoneally with 1% cyclophosphamide (30 mg/kg body weight) once a day for 5 consecutive days. The rats in the QJT group were given a dose of QJT (0.45 g/kg·d) by gavage, while the rats in the Control group and the MO-AT group were given 10 ml/kg normal saline by gavage. Continuous administration was maintained for four weeks. The animals followed the anesthesia procedure, and the anesthetic drug was pentobarbital sodium. Figure 1 description of the experimental process.

### Computer assisted semen analysis (CASA)

The epididymis of rats were cut into small slices in the pHam’s F-10 culture medium and incubated at 37℃ for half an hour [20] to release the spermatozoa into medium. In order to minimize the damage, we use microsurgical scissors to gently cut the epididymal epidermis to avoid damaging the subepidermal tissue of epididymis. After the sperm in epididymis was fully free, the amount of sperm and sperm viability were detected by a digital color sperm quality detection system.

### SOD and MDA evaluation

Commercial enzyme-linked immunosorbent assay (ELISA) kits was used to evaluate the levels of SOD and MDA in testicular tissue according to the protocol set by the respective manufacturers. Read absorbance at 450 nm with a microplate reader (Tecan, Switzerland).

### Histological analysis

The testes were fixed with 10% neutral formaldehyde fixative and embedded in paraffin. Cut the paraffin section to 4 μm, hematoxylin and eosin (H&E) staining were used for histological evaluation according to the standard procedure. Immunohistochemical analysis: The slides were incubated for 16 h at 4 °C with primary antibody of Occludin (abcam) and Zo-1 (abcam), followed by incubation with a secondary antibody for 1 h. The immunostained sections were developed by DAB and haematoxylin staining. Then, scan the stained sections with a light microscope (Leica microscope DM500, Germany).

#### Immunofluorescence assays

Rat testis tissue was fixed by soaking in Bouin fixing fluid at room temperature for 48 h, and they then were dehydrated, embedded in paraffin, and cut into 5-μm-thick sections that were collected on glass slides. After dewaxing and dehydrating the sections, the antigen was retrieved under high pressure. Immerse testicular sections in normal goat serum to block non-specific antigens, and then incubate them with F-actin primary antibody overnight at 4℃. Finally, incubate the sections with a fluorescein-labeled secondary antibody. Positive fluorescence in areas of interest in sections was photographed (Leica microscope DM3000, Germany).

### Western blot (WB) analysis

Equal quantities of protein from the testis tissue lysate were processed for Western blotting, and each sample was denatured, electrophoresed, and transferred onto a polyvinylidenedifluoride membrane. The membrane was blocked with 5% bovine serum albumin and immunolabeled with Anti-p38 (abcam), Anti-F-actin (abcam), and Anti-Occludin (abcam) antibodies, followed by incubation with HRP-labeled anti-rabbit IgG antibody and Clarity Western ECL Substrate. Blots were then stripped with 50 mM glycine and reprobed with HRP-labeled Anti-p38 (abcam), Anti-F-actin (abcam), and Anti-Occludin (abcam) antibodies. The western blotting results include representative images of the blots adjusted according to the contrast and density of each band normalized to p38, F-actin, and Occludin.

#### Statistical analysis

Analysis software: SPSS 22.0. The quantitative experimental data satisfying normal distribution assumptions were analyzed by t-test, and the comparison of mean between groups was analyzed by one-way ANOVA. The quantitative experimental data not satisfying normal distribution assumptions were tested by nonparametric tests. Rank sum testing was used for variance inequality. A chi-squared test was used for rate comparison. All values are shown as mean ± standard deviations. *P* < 0.05 means the difference is statistically significant.

## Results

### Body weight and testicle weight

As shown in Tables 1 and 2, the model rats in MO group showed lower body weight compared to the healthy rats in Control group (*p* < 0.0001). In the intervention period, the weight of rats in the MO-AT group was significantly decreased on the 8^th^, 15^th^, 22^nd^, and 29^th^ days (*P* = 0.0188, *P* = 0.0057, *P* = 0.0002, *P* = 0.0466) compared with the Control group. The changes in body weight were significantly different on the 8^th^, 15^th^, 22^nd^ and 29^th^ days (*P* = 0.0121, *P* = 0.0013, *P* = 0.0073, *P* = 0.0283) between the Control group and the QJT group. There were no significant difference in weight changes between QJT group and MO-AT group. The initial and final right testicle weight were also measured. In the model-making period, the final right testicle weight decreased significantly in the MO group (1.22 ± 0.32 g) in comparison with the control group (1.62 ± 0.07 g) (p = 0.0011). In the intervention period, the final right testicle weight decreased significantly in the Mo-AT group (1.45 ± 0.010 g) and in the QJT group (1.44 ± 0.21 g) compared with the Control group (1.79 ± 0.11 g) (*p* < 0.0001, *p* < 0.0001).

**Table 1:** Body weight in the model-making period (g, $\overline{{\varvec{x}} }$ ± SD)

Group	*n*	Day 1	Day 2	Day 3	Day 4	Day 5
Control	20	203.02 ± 4.56	205.47 ± 6.76	217.47 ± 6.36	223.73 ± 10.13	228.41 ± 7.61
MO	30	203.91 ± 5.03	202.47 ± 6.63	206.19 ± 8.82^*^	201.49 ± 10.07^*^	206.35 ± 13.31^*^

Values are mean ± SD,^*^P < 0.05 vs control group

**Table 2:** Body weight in the intervention period (g, $\overline{{\varvec{x}} }$ ± SD)

Group	*n*	Day0(Before administration)	Day 8	Day 15	Day 22	Day 29
Control	10	224.20 ± 9.23	275.94 ± 22.97	309.19 ± 32.62	328.75 ± 33.47	340.56 ± 30.23
MO-AT	10	224.11 ± 27.89	245.90 ± 26.86^*^	271.56 ± 20.63^*^	273.07 ± 23.74^△^	313.59 ± 25.32^*^
QJT	10	224.75 ± 18.37	244.01 ± 18.56^△^	265.13 ± 18.48^*^	289.96 ± 19.56^*^	308.13 ± 23.49^*^

Values are mean ± SD,^*^P < 0.05 vs control group. ^△^*P* < 0.05 vs control group

### QJT improved the sperm quality of model rats

The epididymal sperm count was markedly lower in the CP-administered rats compared to the healthy control rats (MO-BT vs. Control, *p* < 0.0001; MO-AT vs. Control, *p* = 0.0497). However, sperm count increased in the QJT group compared to the MO-AT group (*p* = 0.0001). As shown in Tables 3 and 4, there were no significant difference in all other sperm parameters among the experimental groups.

**Table 3:** Sperm quality in the model-making period ($\overline{{\varvec{x}} }$ ± SD)

Group	*n*	Sperm count	sperm motility(PR)(%)
Control	10	33.50 ± 14.07	30.17 ± 13.14
MO-BT	10	5.38 ± 3.42^*^	26.99 ± 29.15

Values are mean ± SD,^*^P < 0.05 vs control group

**Table 4:** Sperm quality in the treatment period ($\overline{{\varvec{x}} }$ ± SD)

Group	*n*	Sperm count	sperm motility(PR)(%)
Control	10	80.00 ± 38.83	32.14 ± 9.57
MO-AT	10	59.86 ± 36.43^*^	26.27 ± 12.79
QJT	10	160.86 ± 59.43*^△^	23.14 ± 7.98

Values are mean ± SD,^*^P < 0.05 vs control group.^△^*P* < 0.05 vs Mo-AT group

### QJT enhanced testicular antioxidant capacity

As shown in Table 5, the model rats in the MO-BT and MO-AT groups showed a high level of MDA (MO-BT vs. Control, *p* < 0.0001; MO-AT vs. Control, *p* = 0.0408) and a low level of SOD (MO-BT vs. Control, *p* = 0.0212; MO-AT vs. Control *P* = 0.0315) compared to the levels in the Control group. After treatment, the model rats in the QJT group showed a low level of MDA (QJT vs. MO-AT, *p* = 0.0031; QJT vs. MO-BT *p* < 0.0001) and a high level of SOD (QJT vs. MO-BT, *p* < 0.0001; QJT vs. MO-AT, *p* = 0.0448) compared to the levels in the MO-BT and MO-AT groups. The results indicated that QJT alleviated CP-induced oxidative stress damage of testis.

**Table 5:** Effect of QJT on oxidative stress markers in testes of rats

Group	*n*	SOD(ng/g protein)	MDA(nmol/g protein)
Control	10	47.92 ± 13.14	25.24 ± 4.59
MO-BT	10	28.93 ± 13.24^*^	35.99 ± 4.88^*^
MO-AT	10	33.97 ± 14.07^*^	30.27 ± 2.71^*^
QJT	10	51.03 ± 15.13	22.42 ± 5.83

Values are mean ± SD, in comparison with control or QJT group, ^*^P < 0.05

### QJT improved histopathological features in the model rats

**Fig. 2:**
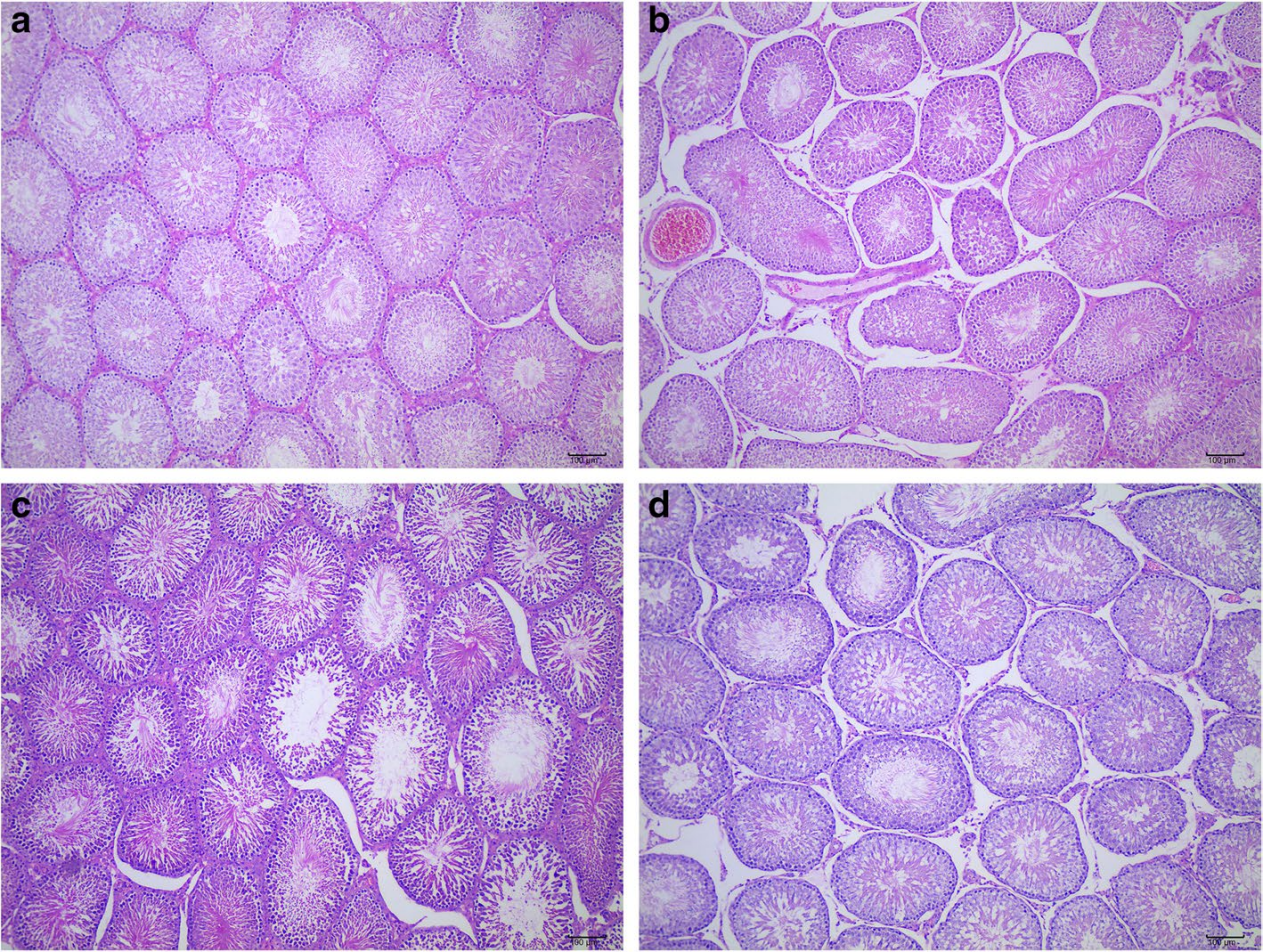
(**a**–**d**) H&E staining images of testicular tissues in (**a**) Control group, (**b**) MO-BT group, (**c**) MO-AT group, and (**d**) QJT group. (Magnification of × 100, Scale Bar = 100 μm)

**Fig. 3:**
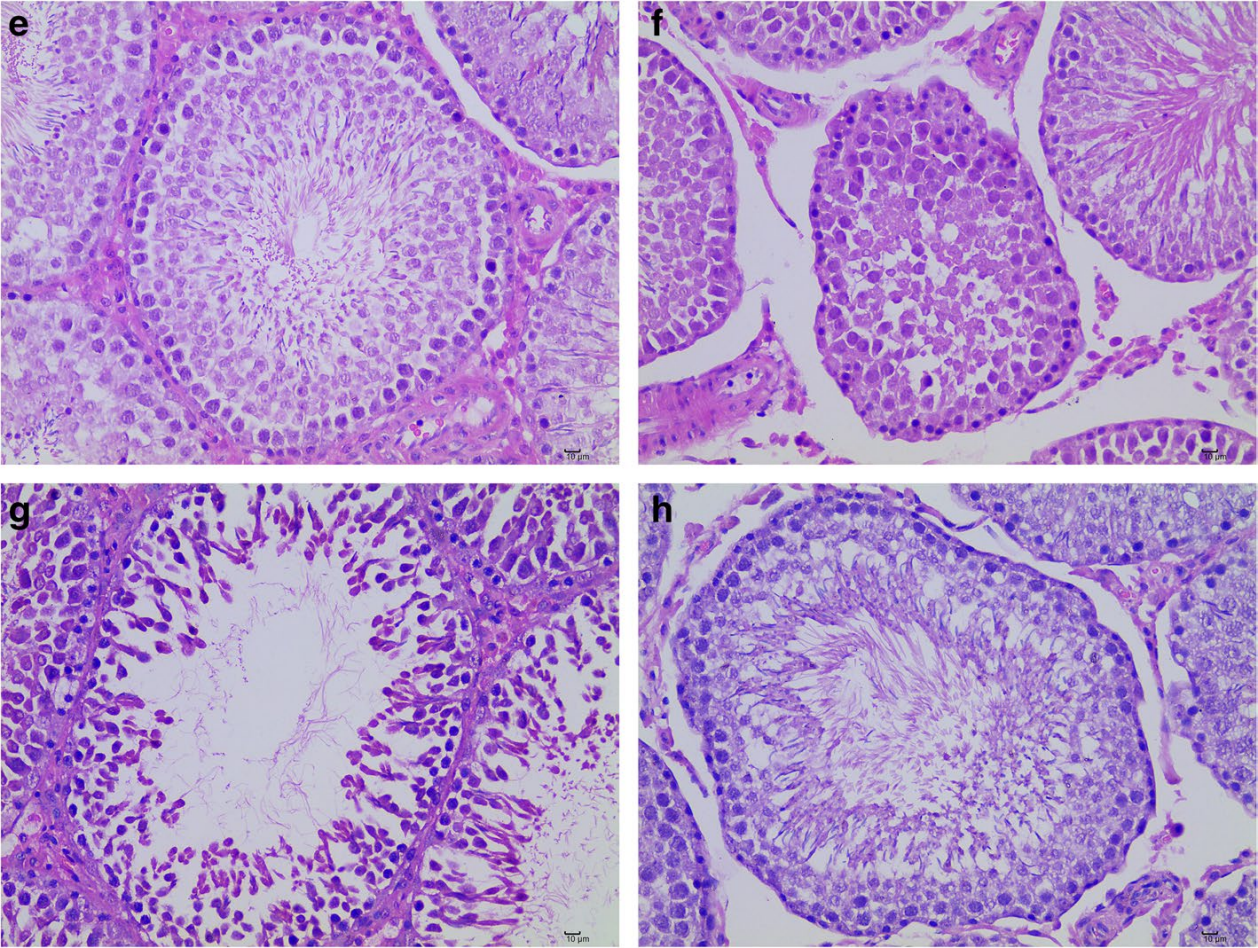
(**e–f**) H&E staining images of testicular tissues in (**e**) Control group, (**f**) MO-BT group, (**g**) MO-AT group, and (**h**) QJT group. (Magnification of × 400,Scale Bar = 10 μm)

Figures 2 and 3 show the testicular H&E staining images of different experimental groups. In Control group, the seminiferous tubules were closely arranged with regular lumens (Magnification of × 100). Spermatogenic epithelium include Sertoli cells, spermatogonia, spermatocytes, and spermatids. The cells were arranged in regular layers, and there were varying amounts of spermatozoa in lumens (Magnification of × 400). In MO-BT group, the lumens of seminiferous tubules were irregular with exfoliation of spermatogenic epithelium (↑) (Magnification of × 100). Loss of spermatozoa and disorder of cells arrangement in the lumens of seminiferous tubules were present (↑) (Magnification of × 400). In MO-AT group, The arrangement of seminiferous tubules were relatively close, the lumens were regular, and the arrangement of seminiferous epithelial cells were loose(↑) (Magnification of × 100).The cell composition was reduced, and the cells were disordered. Necrosis, pyknosis, and even dissolution of the nucleus and spermatozoa deficiency could be seen (↑) (Magnification of × 100). In QJT group, the seminiferous tubules were closely arranged, and most of the seminiferous epithelium were normal (Magnification of × 100). In a few lumens, the cells were disordered (↑). Most of the seminiferous epithelial structures were basically normal, the spermatogenic cells were arranged more orderly, compact and normal in morphology and structure and the quantity of sperms, spermatogenic cells and interstitial cells were increased (Magnification of × 400).

In summary, the spermatogenic epithelial cells in testis were reduced or exfoliated in the MO-BT group. After four weeks of natural recovery, the pathological changes in the MO-AT group were slightly alleviated compared with the MO-BT group. The pathological injury in the QJT group were resolved compared with the MO-BT group. The results showed that QJT ameliorated CP-induced histopathological injury.

### QJT increased Occludin and ZO-1 positive staining

**Fig. 4:**
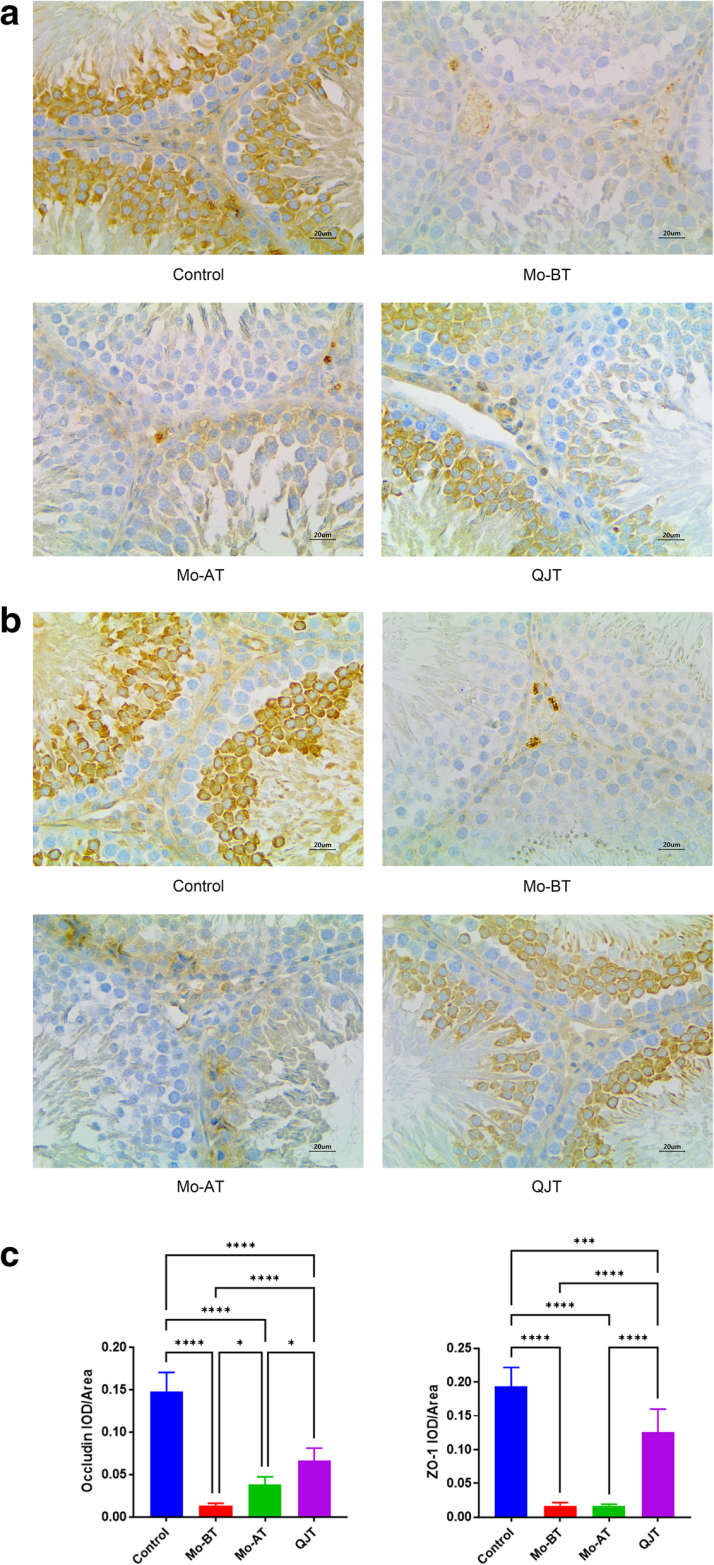
**A** Immunohistochemical staining of occludin in testicular tissues of each group: (**a**) Control group, (**b**) MO-BT group, (**c**) MO-AT group, and (**d**) QJT group. (Scale Bar = 20 μm) 4**B** Immunohistochemical staining of ZO-1 in testicular tissues of each group: (**a**) Control group, (**b**) MO-BT group, (**c**) MO-AT group, and (**d**) QJT group. (Scale Bar = 20 μm) 4**C** Semi quantitiative analysis of immunohistochemistry of Occludin and ZO-1 in testicular tissues of each group: Control group, MO-BT group, MO-AT group, and QJT group.*P < 0.05

Figure 4a-c show the expressions of ZO-1 and Occludin by immunohistochemistry in all groups. In the Control group, the ZO-1 and Occludin positive staining were obvious, while in the MO-BT group, they were decreased clearly. After four weeks of natural recovery, the ZO-1 and Occludin positive staining increased slightly in the MO-AT group. After QJT treatment, the ZO-1 and Occludin positive staining in the QJT group increased significantly compared to the MO-BT group and MO-AT groups. Meanwhile, semi quantitative analysis of immunohistochemistry showed that ZO-1 (Control vs. MO-BT and MO-AT, *P* < 0.0001; Control vs. QJT, *P* = 0.0002; QJT vs. MO-BT and MO-AT, *P* < 0.0001) and Occludin (Control vs. MO-BT, MO-AT and QJT, *P* < 0.0001; QJT vs. Mo-BT, *P* < 0.0001; QJT vs. Mo-AT, *P* = 0.0119) expressions were lower in the MO-BT and MO-AT groups than in the Control and QJT groups. The results showed that CP significantly decreased the expressions of ZO-1 and Occludin in testis, while QJT increased the expressions of ZO-1 and Occludin.

### QJT increased F-actin positive fluorescence intensity

**Fig. 5:**
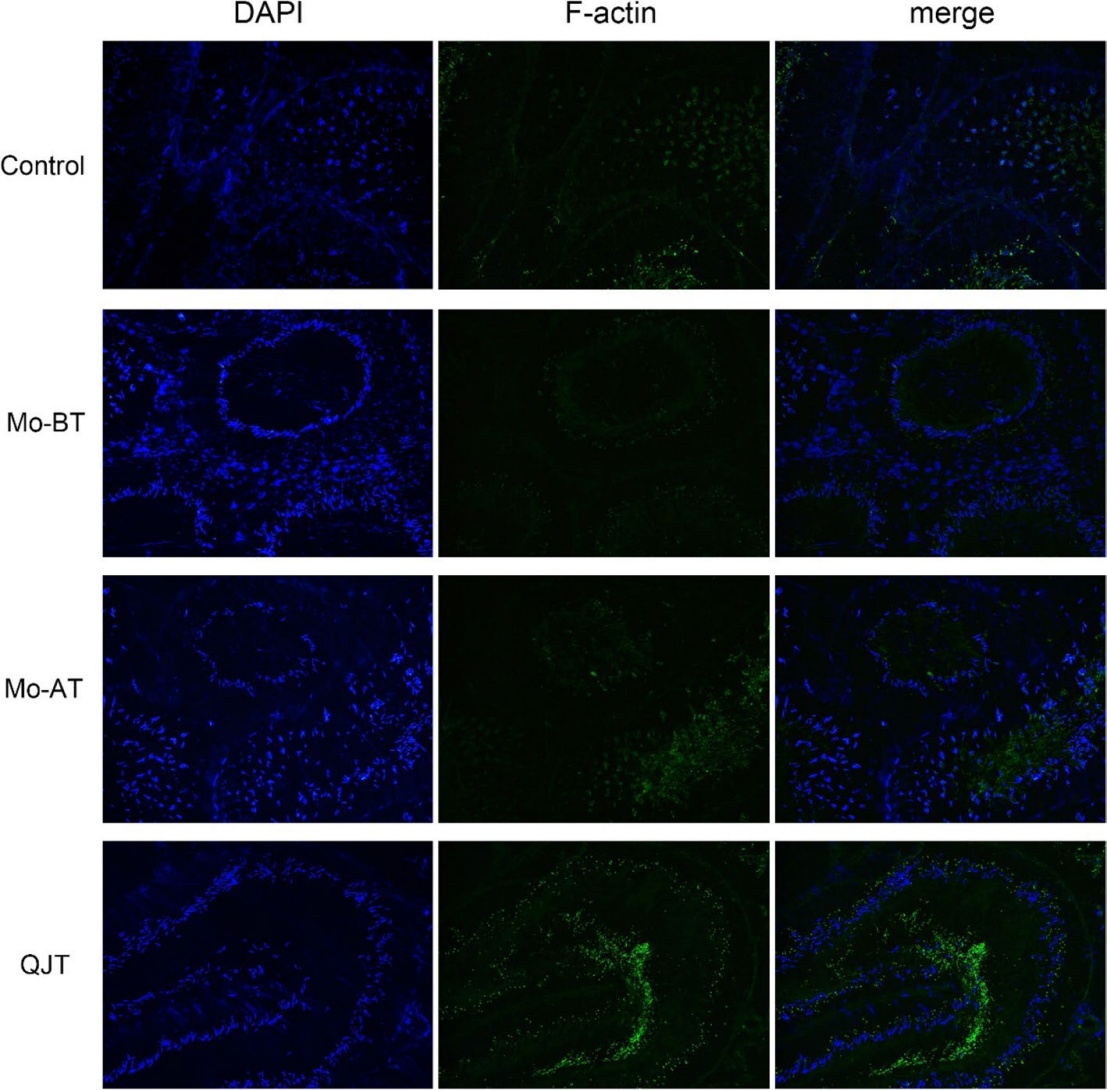
F-actin immunofluorescence in rat testis (200 ×). Fluorescent micrographs using cross sections of deparaffinized testes from rats receiving vehicle: Control group, MO-BT group, MO-AT group, and QJT group. Green fluorescence represents F-actin and DAPI stained for nuclei

Figure 5 shows the expression of F-actin by immunofluorescence in the different experimental groups. The F-actin positive fluorescence intensity (green) decreased significantly in the MO-BT group. After four weeks of natural recovery, the positive fluorescence intensity increased significantly in the MO-AT group. After QJT treatment, the positive fluorescence intensity in the QJT group increased significantly compared to the MO-BT and MO-AT groups. The results indicated that CP significantly decreased the F-actin positive fluorescence intensity, while QJT increased the F-actin positive fluorescence intensity.

### QJT inhibits p38MAPK pathway, increases the expression of BTB related proteins, and repairs the damaged BTB

**Fig. 6:**
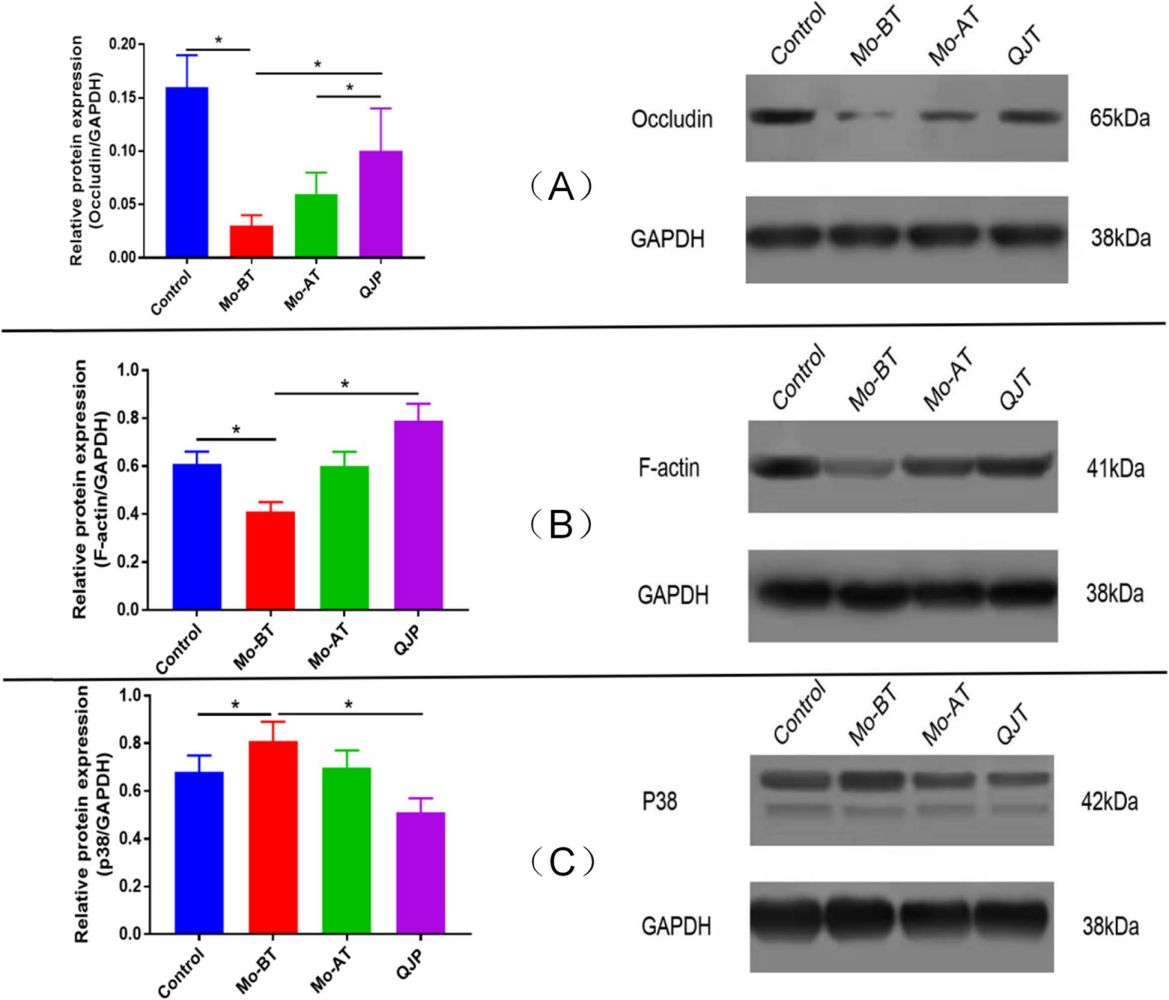
Effects of QJT on the expressions of Occludin, F-actin, and p38 proteins in testicular tissue (Control group, MO-BT group, MO-AT group, and QJT group). (**A**) Western blot (WB) analysis and statistical analysis of the gray value of Occludin activation in the testicular tissue; (**B**) WB analysis and statistical analysis of the gray value of F-actin activation in the testicular tissue; (**C**) WB analysis and statistical analysis of the gray value of p38 protein activation in the testicular tissue. The data indicate the mean ± standard deviation (*n* = 10). *P < 0.05

Figure 6 shows the expressions of Occludin, F-actin, and P38 proteins by WB analysis in the different experimental groups. In testis, the expressions of F-actin and Occludin proteins in the MO-BT group decreased significantly compared to the Control group (*P* = 0.0103, *P* = 0.0018). After four weeks of natural recovery, the expressions of Occludin and F-actin in the MO-AT group were increased, but there were no significant difference compared with MO-BT group. The expressions of Occludin (QJT vs. MO-BT, *P* = 0.0453; QJT vs. MO-AT, *P* = 0.0443) and F-actin (QJT vs. MO-BT, *P* = 0.0002; QJT vs. MO-AT, *P* = 0.0137) in the QJT group increased significantly compared to the MO-BT and MO-AT groups. Meanwhile, the expression of p38 protein in the MO-BT group increased significantly compared to the Control group (*P* = 0.0186). After four weeks of natural recovery, the expression of p38 protein in the MO-AT group was decreased, but there was no significant difference compared with MO-BT group. After QJT treatment, the expression of the p38 protein in the QJT group was significantly lower than that in MO-BT (*P* = 0.0445). The results indicated that QJT inhibited the p38MAPK pathway, increased the expressions of Occludin and ZO-1 proteins, repaired BTB injury induced by CP.

## Discussion

This study explored the possible mechanisms of QJT in improving CP-induced rats with BTB dysfunction. The main findings are as follows: (1) In histopathological studies, QJT ameliorates CP-induced histopathological changes. (2) QJT induces a decrease in MDA and an increase in SOD in testicular tissues of model rats with CP-induced BTB injury. (3) QJT can improve the sperm quality of model rats. (4) QJT administration reduces the over-expression of p38 protein in testicular tissues of model rats. Moreover, the expression levels of ZO-1 and Occludin in testis were significantly up-regulated after QJT treatment. These results indicate that QJT has a protective effect on damaged BTB, which may be related to the inhibition of oxidative stress and p38MAPK pathway.

Previous studies have shown that CP (20 ~ 50 ml/kg/day) intraperitoneal injection for 5 ~ 7 consecutive days can cause oxidative stress injury of testis in mouse [21–23]. Meanwhile, CP has been linked to oligozoospermia and azoospermia, as well as to biochemical and histological changes in testes and epididymis in humans and rats [24–27]. Compared with normal mice testis tissue sections, the cross-sections of CP-treated mice are characterized by empty and atrophied seminiferous tubules [28]. After CP intervention, in many tubules, vacuolization of cytoplasm, disruption of cells and nuclear membranes, or even complete necrotisis at different stages of degradation could be seen [29]. Therefore, we used CP to make testicular oxidative stress injury model. In this study, the histological morphology of testis showed that CP led to necrosis, pyknosis, and even dissolution of the nucleus and spermatozoa deficiency. However, QJT resolved the pathological injury of rats testis induced by CP.

The balance between between oxidants and antioxidants is important to maintain the normal function of BTB. Many studies have shown that endogenous antioxidant enzymes (such as SOD) can convert free radicals or reactive oxygen intermediates into non free radical products, and these enzymes play a key role in antioxidant defense. The major enzymes that can remove ROS in male reproductive organs include SOD and GSH [30–32]. Our previous studies showed that QJT had obvious antioxidant effects. Similar results have been obtained in this study. Moreover, Our results suggested that QJT ameliorated oxidative stress-induced testicular injury. These results demonstrated that QJT protected the integrity of BTB against oxidative stress injury as an exogenous antioxidant.

The BTB, which is essential for normal spermatogenic function of testis, is located near the bottom of seminiferous tubules and divides the epithelium into adluminal and basal. The adluminal compartment is located above the tight junction, communicating with the lumen of the seminiferous tubule, and containing spermatocytes, sperm cells and sperm. The basal compartment is located between the seminiferous epithelial basement membrane and the tight junction, containing spermatogonia. Therefore, the main function of BTB is to separate germ cells from lymphatic system and circulatory system, and to provide immune privilege microenvironment for meiosis with local immunosuppression [33, 34]. The BTB created by TJ, ectoplasmic specializations, desmosomes, and gap junctions that are present between Sertoli cells [4, 35–38]. The TJ are the most important components of the BTB, serving gate and fence functions. The gate function creates a barrier to prevent water, solutes, and other large molecules from passing between the paracellular space, while the fence function is mainly to generate cell polarity, limiting the movement of proteins and lipids between apical and basolateral domains [39]. Proteins, such as Occludin and ZO-1, play a key role in maintaining the normal function of TJ. Inhibition of occludin can lead to the reversible loss of BTB function and germ cells of epithelial cells in rat testis, indicating that occludin plays an important role in BTB [40]. The N-terminal of ZO-1 can directly bind to the C-terminal of Occludin protein and connect with the intracellular skeleton protein to form a stable connection system, which is the structural basis of TJ. Regularly arranged actin filaments, TJ, and spermatogenic cells in testis are important for the overall morphology, Sertoli cells, and the maintenance of the BTB [41]. Occludin is closely related to actin microfilaments. Membrane regulatory proteins, ZO-1, and actin microfilaments participate in the formation of TJ network structures [42, 43]. The fibros actin (F-actin), as an actin filament Polymer, is connected with ZO-1, which ensures the integrity of TJ network structures. In addition, an actin binding protein (ABP), such as Eps8 [44] or Arp3 [45], could regulate the BTB cycle and spermatogenesis, and its regulatory mechanism is related to the regulation of F-actin distribution in Sertoli cells. Therefore, the abnormal expressions of junction proteins in Sertoli cells can increase the permeability of the BTB destroying its structure and function [46, 47]. Studies have confirmed that oxidative stress can cause abnormal expressions of TJ related proteins by activating the p38 MAPK pathway [9, 48]. The activation of oxidative stress/p38 MAPK pathway plays a key role in the destruction of the TJ barrier [49, 50]. In the process of cytokine or cadmium induced BTB recombination or degradation, the activation of the p38 MAPK pathway is related to the destruction of TJ and the loss of TJ proteins [51, 52]. The increase of p38MAPK pathway activity leads to ubiquitination and proteasome degradation of Occludin, and the caveolae dependent endocytosis of junction proteins (N-cadherin) impairs the BTB integrity [53, 54].

In our study, the expressions of Occludin, ZO-1, F-actin and p38 were examined in each group. CP could reduce the expressions of Occludin, ZO-1 and F-actin, and increase the expression of p38 significantly. However, these indicators returned after the treatments with QJT, demonstrating the role of QJT in regulating the expressions of key proteins on BTB and p38 MAPK pathway. Here, we observed the protective effects of QJT in mitigating BTB dysfunction in CP-induced rats. This study revealed part of the possible mechanisms, but further studies are still needed to reveal more evidence.

## Conclusion

QJT could alleviate the pathological injury, upregulate expressions of ZO-1, Occludin, and F-actin to improve the integrity of BTB in CP-induced rats with BTB dysfunction via regulating oxidative stress and p38 MAPK pathway.

## Supplementary Information

**Additional file 1**.

## Data Availability

All data generated or analyzed during this study are included in this published article [and its supplementary information files].
