# Supplementary material for: Qiangjing tablets repair of blood-testis barrier dysfunction in rats via regulating oxidative stress and p38 MAPK pathway
Source: BMC Complement Med Ther. 2022 May 14;22:133. doi: 10.1186/s12906-022-03615-z (PMC9107122; doi:10.1186/s12906-022-03615-z)
Supplement: Supplementary file 1 — Additional file 1. [file 12906_2022_3615_MOESM1_ESM.doc]

**Figure**
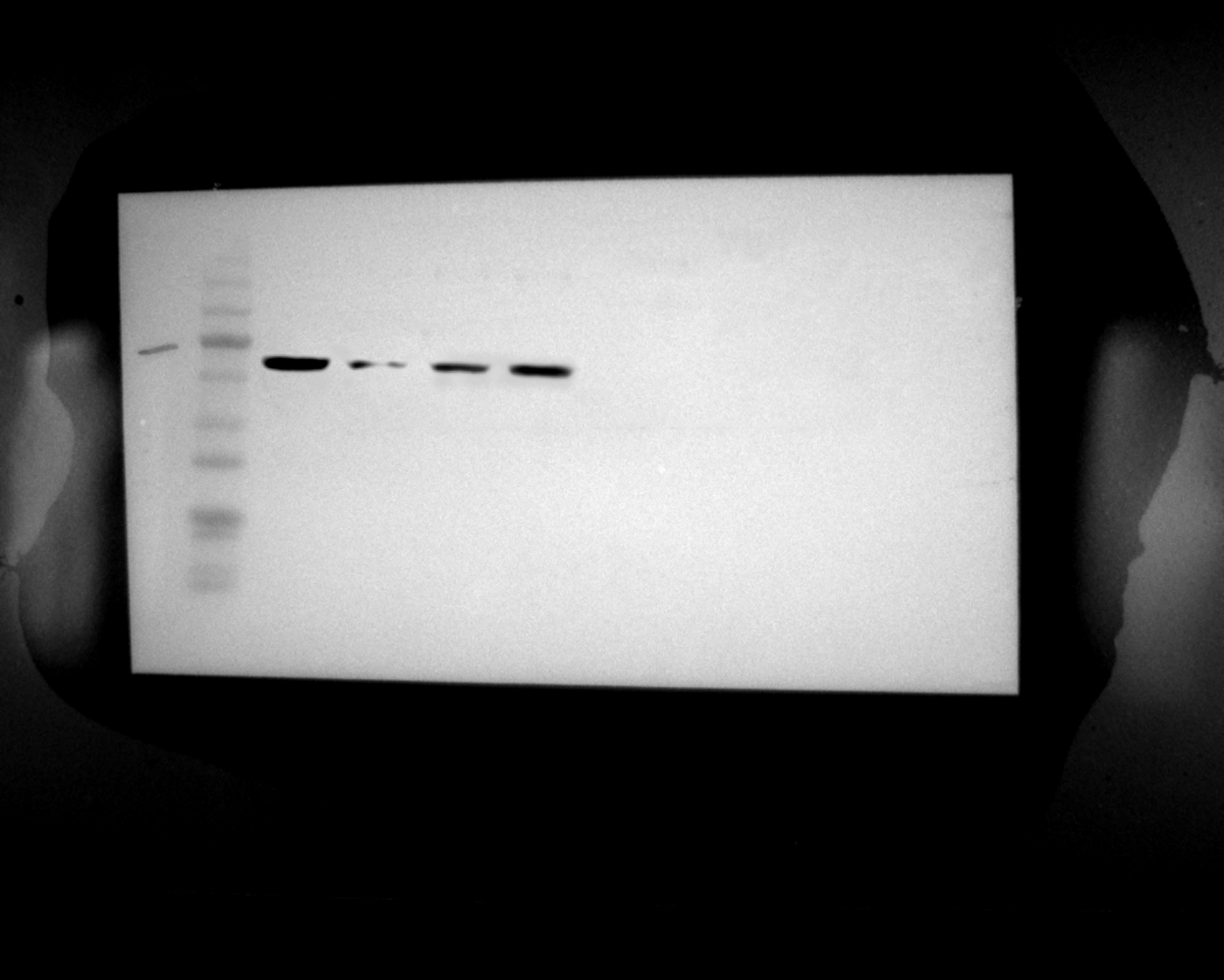
**6(A)-1** Original WB of Occludin in testicular tissues of each group. QJT increased the expression of Occludin in testis of model rats. (From left to right are Control group, MO-BT group, MO-AT group and QJT group)

**
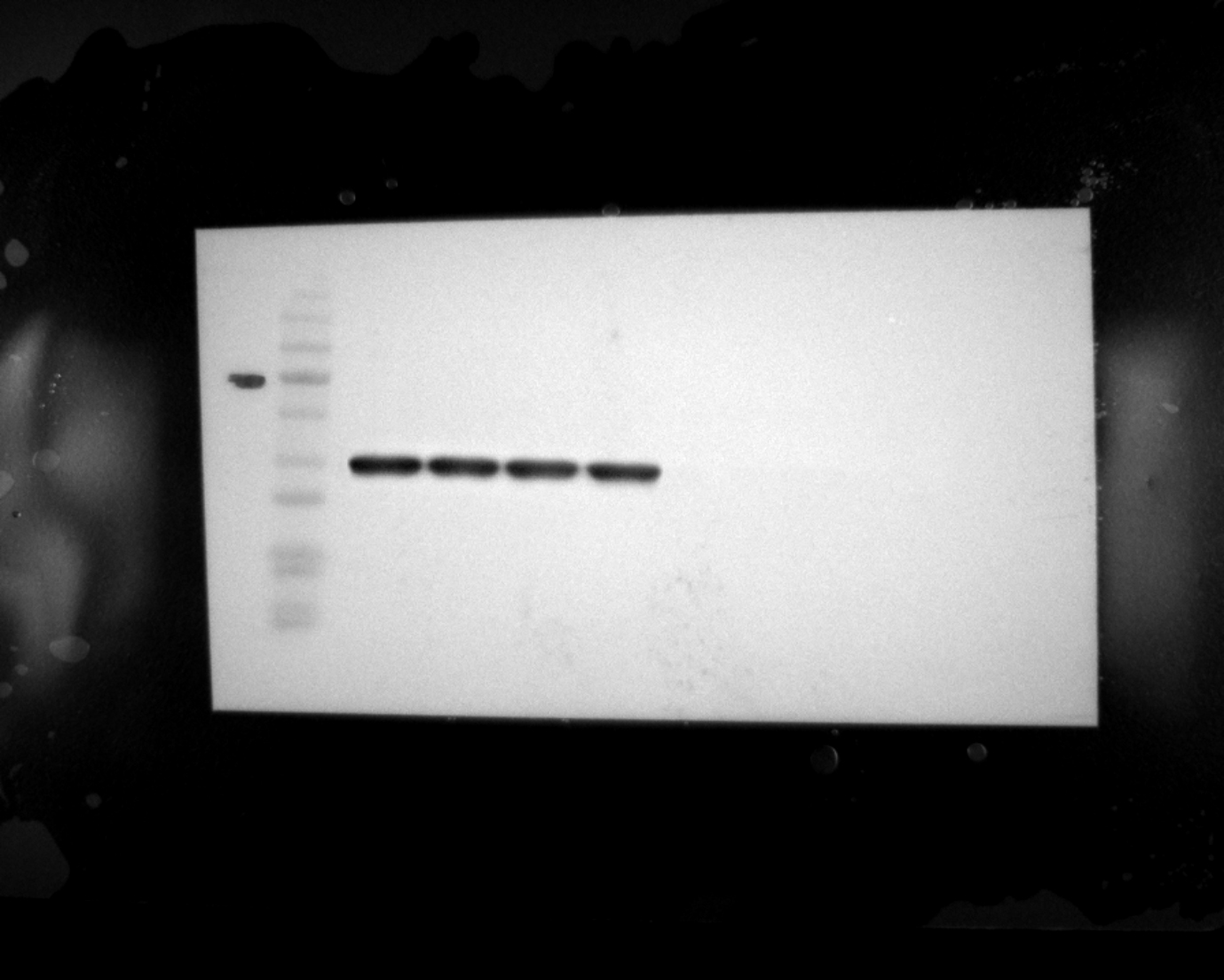
**

**Figure 6(A)-2** Original WB of Occludin-GAPDH in testicular tissues of each group. (From left to right are Control group, MO-BT group, MO-AT group and QJT group)

**
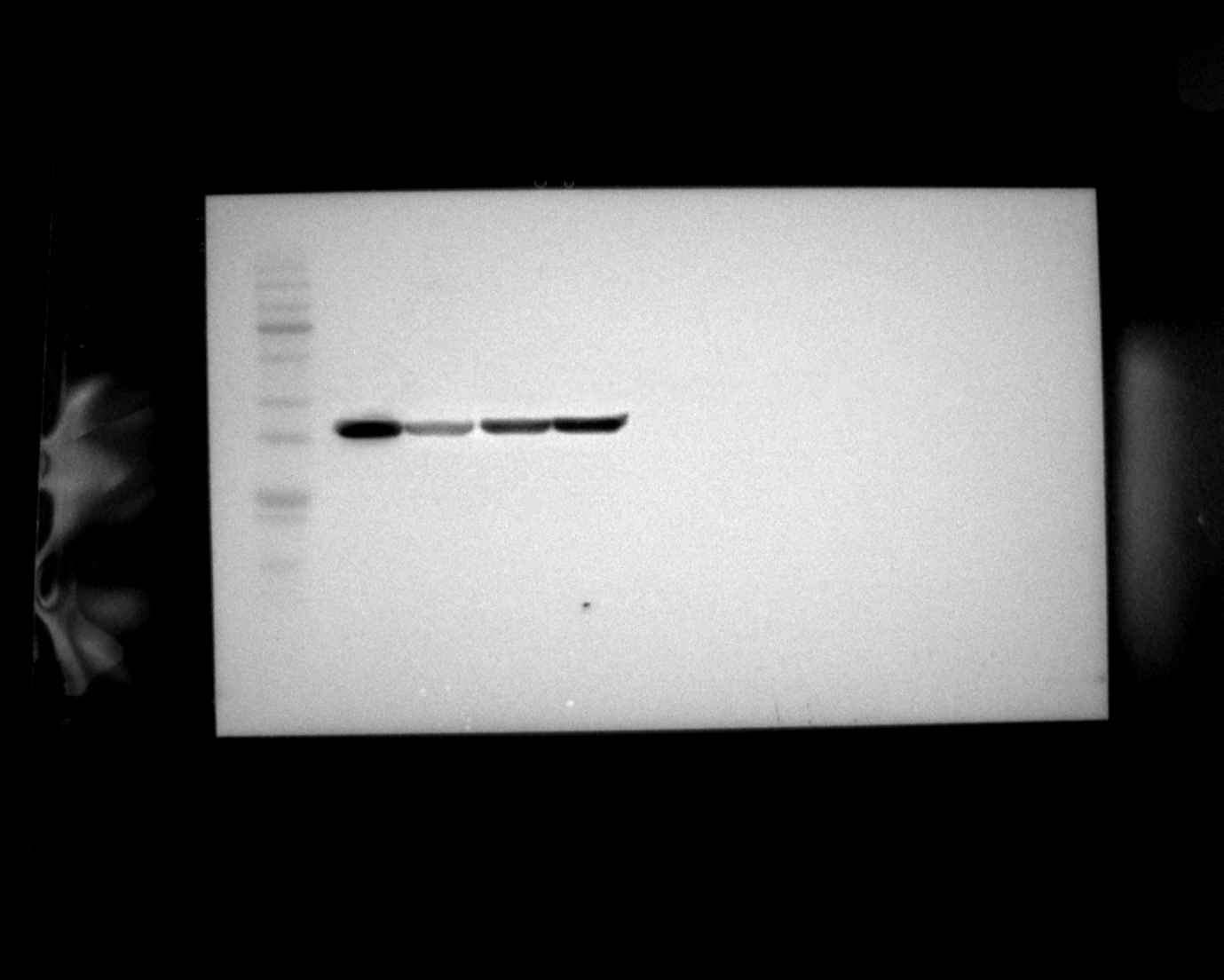
**

**FIGURE 6(B)-1** Original WB of F-actin in testicular tissues of each group. QJT increased the expression of F-actin in testis of model rats. (From left to right are Control group, MO-BT group, MO-AT group and QJT group)

**
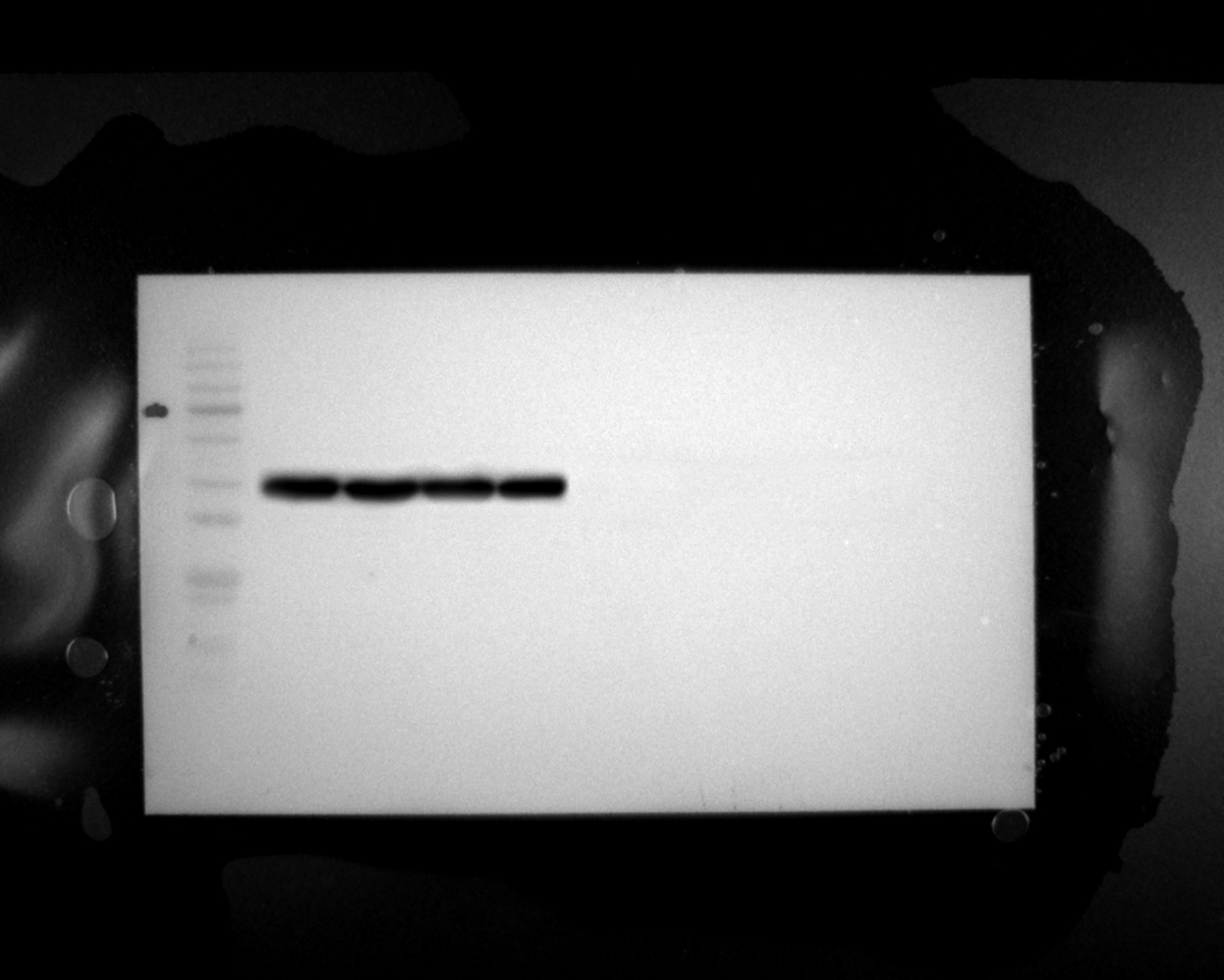
**

**FIGURE 6(B)-2** Original WB of F-actin-GAPDH in testicular tissues of each group. (From left to right are Control group, MO-BT group, MO-AT group and QJT group)


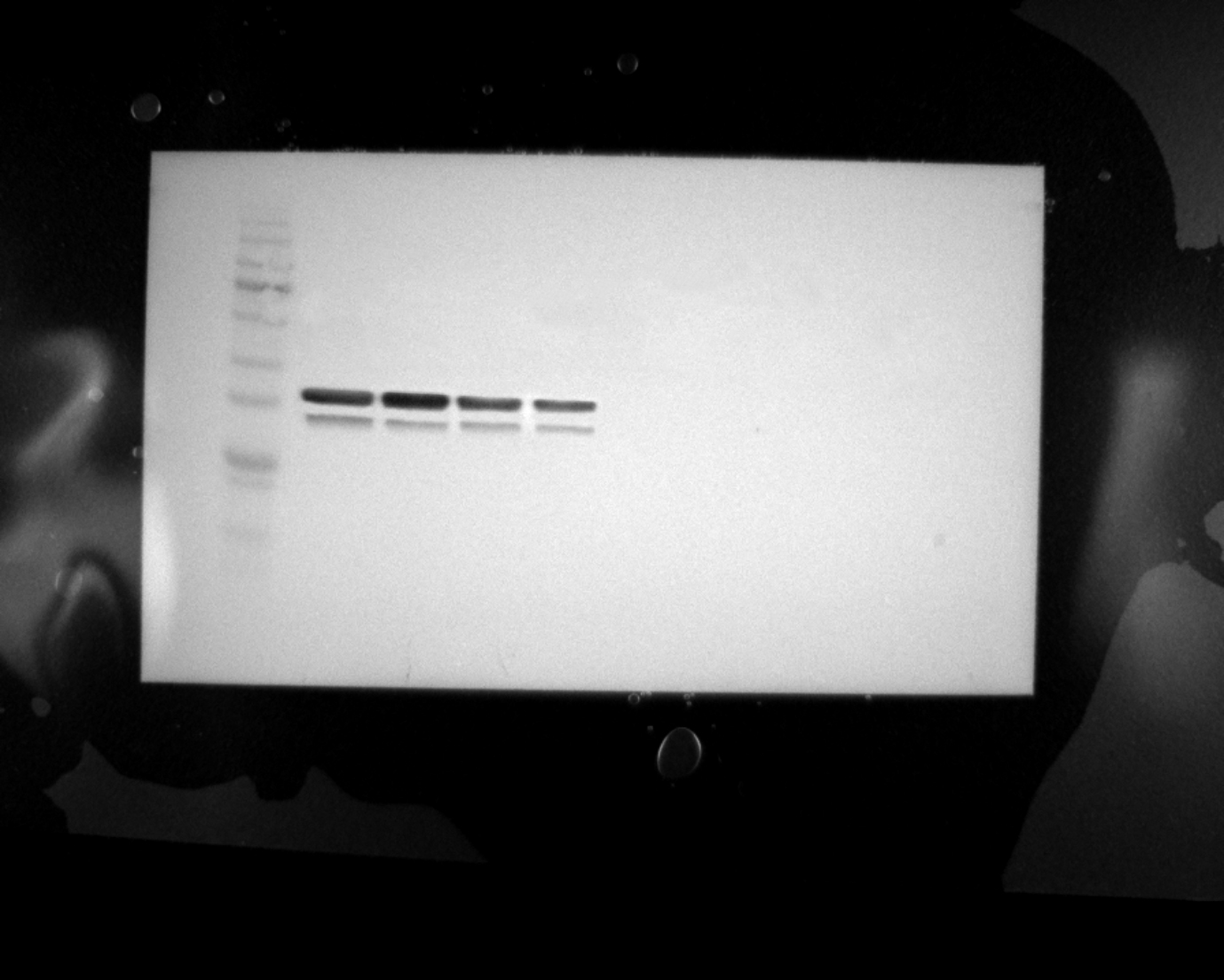


**FIGURE 6(C)-1** Original WB of P38 in testicular tissues of each group. QJT decreased the expression of P38 in testis of model rats. (From left to right are Control group, MO-BT group, MO-AT group and QJT group)


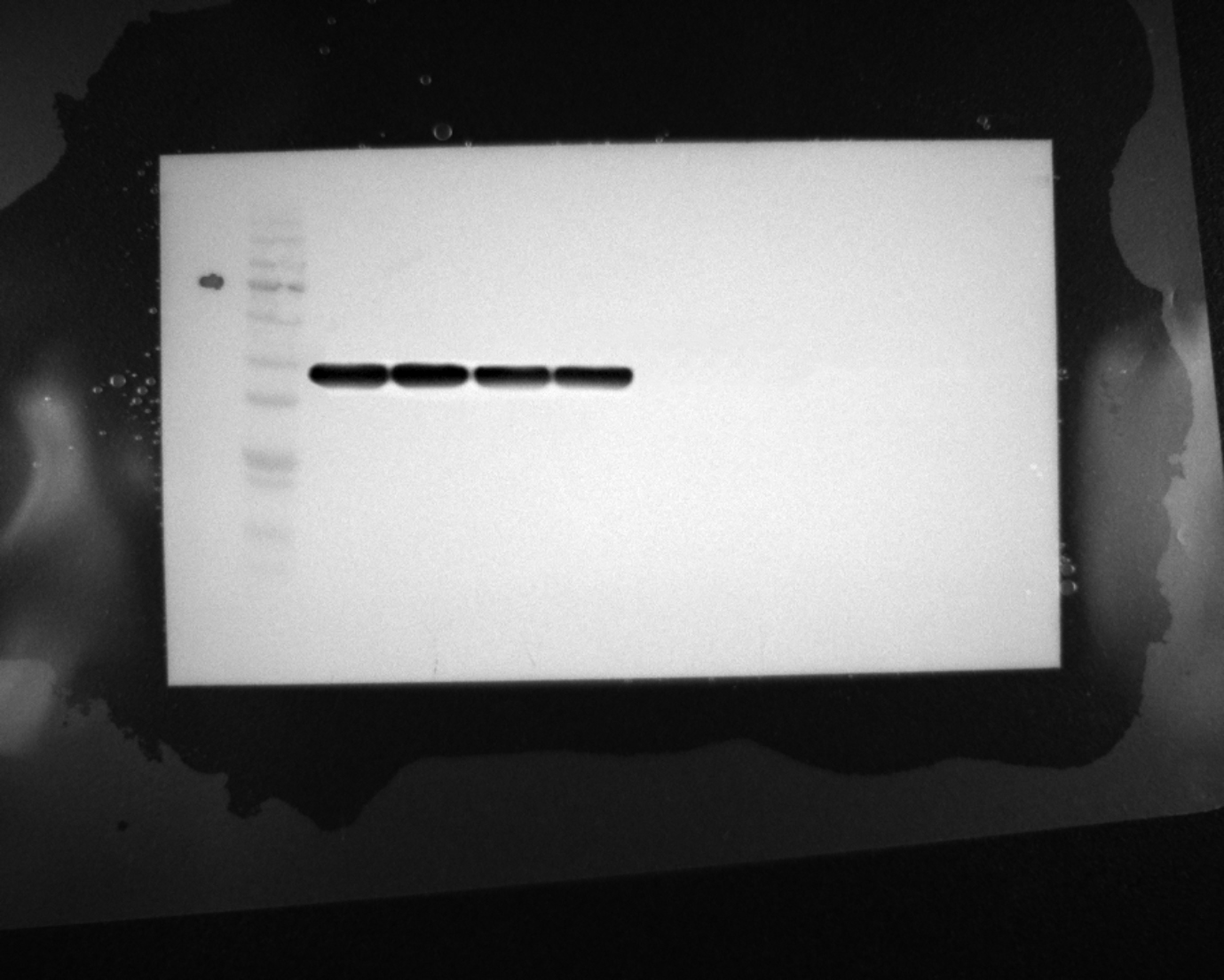


**FIGURE 6(C)-2** Original WB of P38-GAPDH in testicular tissues of each group. (From left to right are Control group, MO-BT group, MO-AT group and QJT group)
